# Enzymatic Shaving of the Tegument Surface of Live Schistosomes for Proteomic Analysis: A Rational Approach to Select Vaccine Candidates

**DOI:** 10.1371/journal.pntd.0000993

**Published:** 2011-03-29

**Authors:** William Castro-Borges, Adam Dowle, Rachel S. Curwen, Jane Thomas-Oates, R. Alan Wilson

**Affiliations:** 1 Department of Biology, University of York, Heslington, York, United Kingdom; 2 Department of Biology and Centre of Excellence in Mass Spectrometry, Technology Facility, University of York, Heslington, York, United Kingdom; 3 Department of Chemistry and Centre of Excellence in Mass Spectrometry, University of York, Heslington, York, United Kingdom; James Cook University, Australia

## Abstract

**Background:**

The membrane-associated and membrane-spanning constituents of the *Schistosoma mansoni* tegument surface, the parasite's principal interface with the host bloodstream, have recently been characterized using proteomic techniques. Biotinylation of live worms using membrane-impermeant probes revealed that only a small subset of the proteins was accessible to the reagents. Their position within the multilayered architecture of the surface has not been ascertained.

**Methodology/Principal Findings:**

An enzymatic shaving approach on live worms has now been used to release the most accessible components, for analysis by MS/MS. Treatment with trypsin, or phosphatidylinositol-specific phospholipase C (PiPLC), only minimally impaired membrane integrity. PiPLC-enriched proteins were distinguished from those released in parasite vomitus or by handling damage, using isobaric tagging. Trypsin released five membrane proteins, Sm200, Sm25 and three annexins, plus host CD44 and the complement factors C3 and C4. Nutrient transporters and ion channels were absent from the trypsin fraction, suggesting a deeper location in the surface complex; surprisingly, two BAR-domain containing proteins were released. Seven parasite and two host proteins were enriched by PiPLC treatment, the vaccine candidate Sm29 being the most prominent along with two orthologues of human CD59, potentially inhibitors of complement fixation. The enzymes carbonic anhydrase and APD-ribosyl cyclase were also enriched, plus Sm200 and alkaline phosphatase. Host GPI-anchored proteins CD48 and CD90, suggest ‘surface painting’ during worm peregrination in the portal system.

**Conclusions/Significance:**

Our findings suggest that the membranocalyx secreted over the tegument surface is not the inert barrier previously proposed, some tegument proteins being externally accessible to enzymes and thus potentially located within it. Furthermore, the detection of C3 and C4 indicates that the complement cascade is initiated, while two CD59 orthologues suggest a potential mechanism for its inhibition. The detection of several host proteins is a testimonial to the acquisitive properties of the tegument surface. The exposed parasite proteins could represent novel vaccine candidates for combating this neglected disease.

## Author Summary

Adult schistosome parasites can reside in the host bloodstream for decades surrounded by components of the immune system. It was originally proposed that their survival depended on the secretion of an inert bilayer, the membranocalyx, to protect the underlying plasma membrane from attack. We have investigated whether any proteins were exposed on the surface of live worms using incubation with selected hydrolases, in combination with mass spectrometry to identify released proteins. We show that a small number of parasite proteins are accessible to the enzymes and so could represent constituents of the membranocalyx. We also identified several proteins acquired by the parasite on contact with host cells. In addition, components of the cytolytic complement pathway were detected, but these appeared not to harm the worm, indicating that some of its own surface proteins could inhibit the lytic pathway. We suggest that, collectively, the ‘superficial’ parasite proteins may provide good candidates for a schistosome vaccine.

## Introduction

The persistence of adult schistosomes in the bloodstream for decades means they must deploy unique and effective immune evasion strategies at their interface with the host. The 1 cm-long worms are covered by a naked syncytial layer of cytoplasm, the tegument, connected by cytoplasmic tubules to underlying cell bodies that contain the machinery for protein synthesis, packaging and export. The tegument surface has a multilaminate appearance, interpreted as a plasma membrane overlain by a lamellate secretion, the membranocalyx [1]. This complex molecular architecture is maintained by export of the contents of multilaminate vesicles, which originate in the cell bodies of the syncytium. There is experimental evidence for slow turnover of the membranocalyx to the external environment [2] whilst recycling of the plasma membrane by internalisation has been anticipated but not conclusively demonstrated [3]. Initial observations suggested the membranocalyx was an amphipathic bilayer, probably composed of phospholipids, which served as a physical barrier to prevent antibody binding or host leukocyte attachment to the underlying plasma membrane. In addition, its supposed hydrophobic properties coincided with a demonstrable ability of worms in the bloodstream to acquire host molecules, particularly erythrocyte glycolipids (the so-called host antigens) [4]. Whether this acquisition is a deliberate process that benefits the parasite or an accidental consequence of the membranocalyx properties, and any relevance it has to immune evasion, remain unclear.

Building on techniques developed in 1980s to detach the tegument by freeze-thaw and enrich the surface membrane complex by differential centrifugation [5], we have characterized its composition using proteomic techniques [6], [7]. We developed a differential extraction scheme for the membrane preparation, with chaotropic agents of increasing strength, which enabled us to identify both membrane-associated and membrane-spanning constituents. These compositional findings demonstrated the importance of the tegument for nutrient uptake and maintenance of solute balance, as well as the presence of several hydrolases in the surface layers [6]. In a second study we incubated live worms with membrane-impermeant probes to biotinylate the most externally-accessible proteins and then recovered the tagged molecules by affinity chromatography for MS/MS identification [7]. This approach revealed that only a small subset of transporters, membrane structural proteins, enzymes, and schistosome-unique proteins were labelled, together with host immunoglobulins and complement C3. We concluded that these represented the most “exposed” surface constituents but we could not place them within the multilayered architecture of the surface with any certainty.

To add another dimension to our understanding of tegument surface organization we have now used an enzymatic shaving approach on live worms to release the components most accessible to the selected enzymes, for MS/MS analysis. By analogy with techniques for stripping adherent cells from culture flasks, we used trypsin to cleave exposed protein loops or domains without impairing membrane integrity. We also incubated worms with phosphatidylinositol-specific phospholipase C (PiPLC) to release any externally accessible GPI-anchored proteins. As a control for proteins released by the vomiting of gut contents during the incubation period, or damage due to handling, we compared protein release +/− PiPLC, using the iTRAQ technique. Finally we used a phospholipase A2 (PLA2) preparation purified from snake venom to erode the lipid bilayer complex to determine if proteins could be selectively detached. We report that both trypsin and PiPLC removed a small subset of proteins whilst inflicting minimal damage on the worms whereas PLA2 was more destructive. We show that the iTRAQ technique identified both parasite and host proteins enriched by PiPLC treatment whereas trypsin released a different subset, with only Sm200 common to both. The proteins we have identified in the adult are also present as transcripts in the lung schistosomulum. We suggest that collectively they may be candidates for a schistosome vaccine, especially if responses can be targeted to the lungs to interfere with intravascular migration of incoming larvae.

## Methods

### Ethics statement

The procedures involving animals were carried out in accordance with the UK Animals (Scientific Procedures) Act 1986, and authorised on personal and project licences issued by the UK Home Office. The study protocol was approved by the Biology Department Ethical Review Committee at the University of York.

### Parasite maintenance and worm recovery

A Puerto Rican isolate of *S. mansoni* was maintained using albino *Biomphalaria glabrata* snails and NMRI strain mice as laboratory hosts. All animal experiments were approved by the Ethical Review Process Committee of the Department of Biology, University of York. Adult parasites were obtained by portal perfusion of mice seven weeks after exposure to 200 cercariae, using RPMI1640 medium (minus phenol red) buffered with 10 mM HEPES (both from Invitrogen, Paisley, UK). Parasites were extensively washed in the same medium and tissue debris and any damaged individuals removed under a dissecting microscope. No attempt was made to separate males from females.

### Trypsin treatment of live parasites and recovery of peptides

Approximately 800 freshly perfused parasites from 20 mice were used in each of four replicate experiments with trypsin as the shaving enzyme. They were incubated in a 30 mL Corning flask (Corning, NY, USA) containing 5 mL of buffered RPMI, with trypsin MS (Promega, Southampton, UK) added at 10 µg/mL, for 30 min at room temperature (RT). The supernatant was recovered, transferred to a 15 mL Falcon tube and centrifuged at 500×*g* for 30 min to remove any insoluble material such as the haematin particles in gut vomitus. Streptomycin and penicillin were then added to a final concentration of 100 µg/mL to prevent microbial growth and tryptic digestion was continued overnight at 37°C, after which peptides were reduced and alkylated. Reduction was performed in the presence of 20 mM DTT for 30 min at 65°C in a water bath and, after cooling, alkylation was performed in the presence of 80 mM iodoacetamide for 1 h at RT in the dark. Trifluoroacetic acid (TFA) was then added to a final concentration of 0.1% before recovery of peptides by passage through a solid phase Strata C18-E extraction cartridge (55 µm, Phenomenex, Macclesfield, UK), followed by several column washes in 0.1% TFA and final elution in 750 µL of 50% acetonitrile/0.1% TFA. The eluted fraction was concentrated under vacuum to dryness and peptides resuspended in 20 µL 0.1% TFA.

### LC-MS/MS

A 3 µL aliquot of the tryptic peptide preparation was injected onto a reversed-phase PS-DVB monolith column (200 µm i.d.×5 cm, LC Packings, Amsterdam, Netherlands). Peptides were separated using a two-step linear gradient of 2–31.4% (v/v) acetonitrile in 0.1% aqueous heptafluorobutyric acid over 60 min, followed by 31.4–51% (v/v) in the same solvent over 5 min, at a flow rate of 3 µL/min; UV absorbance at 214 nm was monitored. Fractions were collected onto a MALDI target plate using a Probot (Dionex, Bannockburn, USA) with simultaneous addition of matrix solution (6 mg/mL α-cyano-4-hydroxycinamic acid (CHCA, Sigma, Poole, UK) in 60% (v/v) acetonitrile).

Positive-ion MALDI mass spectra (MS) were obtained using a 4700 Proteomics Analyzer with TOF-TOF Optics (Applied Biosystems, Framingham, USA) in reflector mode, over the *m/z* range 800–4000 and monoisotopic masses obtained from centroids of raw, unsmoothed data. The precursor mass window was set to a relative resolution of 50, and the metastable suppressor was enabled. The default calibration was used for MS/MS spectra, which were baseline-subtracted (peak width 50) and smoothed (Savitsky-Golay with three points across a peak and polynomial order 4); peak detection used a minimum S/N of 5, local noise window of 50 *m/z*, and minimum peak width of 2.9 bins. The twenty strongest peaks from each fraction, having a signal to noise (S/N) greater than 50 and a fraction-to-fraction precursor exclusion of ±0.2 Da, were selected for CID-MS/MS analysis. Singly-charged peptides were fragmented with Source 1 collision energy of 1 keV, and air as the collision gas. Peak lists from the MS/MS data, containing all *m/z* values from *m/z* 20 to the precursor *m/z* - 60, with a minimum S/N of 10, were provided by TS2 software (version 1.0.0, Matrix Science Ltd., London, UK). Each list, corresponding to one MALDI plate, was then submitted to a local copy of the Mascot program (version 2.1, Matrix Science) and searched against the SmGenesPlusESTs (260448 sequences; 71029272 residues), an in-house database derived from the publically available data in http://www.genedb.org/genedb/smansoni/), and the NCBInr *Mus musculus* database (139457 sequences) for host proteins. Search parameters specified only tryptic cleavages and allowed for up to one missed site, the variable carbamidomethylation of cysteines, and oxidation of methionines; precursor and product ion mass error tolerance was set to ±0.3 Da. A decoy database, generated by Mascot, was used with the significance threshold for protein identification set to achieve a false positive rate of 1 to 2% and peptide threshold set to ‘least identity’. A protein was considered positively identified if the ion score for a particular peptide had an expect value less than 0.05.

### Phosphatidylinositol-phospholipase C (PiPLC) treatment of live parasites

GPI-anchored proteins were recovered from live worms by *in vitro* incubation with PiPLC as the shaving enzyme, the experiment being performed twice to provide biological replicates. Downstream analysis required the worms from 40 mice, which were perfused and treated in two separate batches to minimise the time *ex vivo*; supernatants were then combined. Each batch was incubated at 37°C for 1 h in the presence of PiPLC (from *Bacillus cereus,* Sigma) at 1.25 Units/mL, with conditions as for trypsin. The supernatant was removed and concentrated at 4°C using a 5000 Da cut-off centrifugation device (Vivaspin 6, West Sussex, UK). The control for secretion, vomitus production and parasite damage due to handling comprised an identical experiment, minus PiPLC. This also served as a ‘background’ control for the other enzyme treatments. For one experiment, PiPLC-released proteins were obtained using PiPLC from a different source (from *Bacillus thuringiensis,* Europa Bioproducts, Wicken, Cambridigeshire, UK) employing the same conditions as above. On that occasion the GPI-released fraction was used for a 2-DE separation.

### Fractionation of the PiPLC-released material

The composition of released material was evaluated by 1-DE using a pre-cast NUPAGE 4–12% Bis-Tris gel (Invitrogen) after a 45 min run at 200 V. The gel was then fixed in 40% methanol, 10% acetic acid for 30 min, stained with SYPRO Ruby (Invitrogen) for 2 h in the dark and imaged using a Molecular Imager FX (Bio-Rad, Bath, UK). Protein content in each lane of the gel was then estimated by densitometric analysis using Quantity One software (Bio-Rad). Fifty µg of the PiPLC-treated sample was also evaluated by mini 2-DE essentially as previously described [8], [9]. After electrophoresis the gel was first stained with SYPRO Ruby, imaged as above, and restained with Bio-Safe Coomassie (BioRad); all visible spots were selected for “in gel” digestion [8]. An aliquot of 1–2 µL of the digestion supernatant containing the peptides was spotted on a MALDI plate and dried before the addition of 0.6 µL of a saturated solution of CHCA matrix (in 50% acetonitrile/0.1% TFA). Peptide fragmentation data from each gel spot was processed by GPS Explorer Software (Applied Biosystems) underpinned by Mascot (settings as above), to provide a putative identity for the protein.

### iTRAQ labelling of peptides

The relative composition of PiPLC-treated and control samples was characterized using isobaric tagging (the iTRAQ labelling technique) following the protocol provided by the manufacturer (Applied Biosystems). Prior to labelling, two aliquots of treated and control samples containing 10 µg protein were taken to provide technical replicates. Briefly, 10 µg of both control and PiPLC-treated samples were individually denatured, reduced, and alkylated with reagents supplied in the iTRAQ kit. Peptides were generated by trypsin digestion using a 1∶20 enzyme/protein ratio, at 37°C for 24 h, and labelled with iTRAQ reagents at lysine, terminal amine groups and partially at tyrosine residues. Test samples were labelled with tags 116 or 117 and control samples with tags 114 or 115, respectively. A peptide mixture was made by combining the four tagged samples and cleaned up using a strong cation-exchange cartridge to remove the detergents and excess iTRAQ reagents. The peptides were then affinity-purified in a Strata C18-E cartridge, eluted as for tryptic peptides, dried using a vacuum concentrator and resuspended in 10–20 µL of 0.1% TFA. LC-MS/MS was performed as described above. Protein identification and peptide quantification was achieved by submitting the TS2-generated MS/MS raw data files to Mascot, searching against SmGenesPlusESTs and NCBInr databases. Search parameters were tryptic peptides, with 0–1 missed cleavage; fixed modifications, β-methylthiolation of cysteines, iTRAQ tagging of lysines and N-terminal amine groups; variable modifications were oxidation of methionines and iTRAQ tagging of tyrosines. Precursor and product ion mass error tolerance was set to ±0.3 Da.

The 114-tagged sample (C1) was taken as the reference for calculating ratios. The Mascot software displays the median normalised geometric mean ratios and a factor from which the geometric standard deviation can be derived. Automatic outlier removal was performed by the Mascot software. A protein was considered enriched if the mean T1/C1 and/or T2/C1 ratios minus the 95% confidence limit exceeded the corresponding C2/C1 ratio plus its 95% confidence limit. For single peptide identifications, a protein was considered enriched if it appeared in both biological replicate experiments, and had a mean treated/control ratio >2.

### Phospholipase A2 (PLA2) treatment of live parasites

The final approach for recovery of parasite surface molecules using enzymatic shaving, involved the treatment of live parasites with PLA2. This enzyme isolated from the venom of *Crotalus durissus terrificus* (deposited under accession number P24027 at NCBInr) was kindly provided by Prof. Andreimar Soares (Faculty of Pharmaceutical Sciences, University of Sao Paulo, Brazil). During the shaving experiment, approximately 800 freshly perfused parasites were incubated in a 30 mL Corning flask, containing 6 mL of buffered RPMI1640, with PLA2 added at 16 µg/mL for 1 h, at RT. Another batch of parasites was used in a control and parallel experiment in which no enzyme was added to the culture medium. The supernatants from control and treated samples were concentrated using a Vivaspin 6 filtration device (5000 Da cut off) at 500×*g*, at 4°C until the volume reached 500 µL. The samples were then transferred to 1.5 ml eppendorf tubes and centrifuged at 25,000×*g* for 20 min. After this step a membranous pellet, recovered from PLA2-treated parasites only, was extracted in 50 µL of 0.5% Triton-X100, yielding approximately 20 µg of protein. Reduction, alkylation, trypsin digestion and peptide clean-up were performed as described for the iTRAQ protocol (omitting the labelling steps). LC-MS/MS of the peptide mixture was performed essentially as described above.

### Immunocytochemical localization of host membrane proteins by confocal microscopy

Host plasma membrane proteins on the surface of adult parasites were investigated either on live worms, perfused from mice using RPMI medium, or by preparing OTC-embedded cryostat sections. Both were incubated with primary antibodies at 1∶100 dilution in PBS for parasite sections and in RPMI for live worms, containing 5% normal goat serum, for 1 h at RT. Monoclonal antibodies used were rat anti-mouse CD44 (558739), rat anti-mouse CD90 (Thy-1; 553016) and hamster anti-mouse CD48 (553682), all from BD Biosciences Pharmingen, New Jersey, USA. Labelling was detected by the use of goat anti-rat IgG conjugated to Alexa fluor 488 at 1∶500 dilution for 30 min in the same buffer, or goat anti-hamster IgG conjugated to Alexa fluor 568 under the same conditions.

### Transcript searching

A searchable database was created in 2005, comprising all *S. mansoni* transcripts then available from dbEST (http://www.ncbi.nlm.nih.gov/), the Sao Paulo *Schistosoma mansoni* EST genome project (http://bioinfo.iq.usp.br/schisto/), and the Wellcome Trust Sanger Institute ftp site (ftp://ftp.sanger.ac.uk/pub/pathogens/Schistosoma/mansoni/ESTs). All proteins of interest identified by the enzymatic shaving approach were searched against the compiled EST data to determine the number of transcripts detected in adults and lung stage schistosomula; this provided a very approximate guide to the relative levels of expression in the two life cycle stages.

## Results

### Enzymatic shaving using MS grade trypsin

Freshly perfused live parasites were subjected to trypsin digestion under controlled conditions, in order to release from the surface membrane complex of the tegument any proteins, or segments thereof, accessible to the enzyme. The vast majority of worms retained a normal appearance and activity over the 30 min incubation. The culture supernatant was recovered and released proteins/peptides allowed to digest further overnight. Tryptic peptides were then reduced and alkylated before their recovery and separation using reversed-phase chromatography (Figure S1A) for subsequent mass-spectrometric identification. The identities obtained by Mascot searching of the MS/MS data were then categorized according to their molecular function and, by inference, their potential cellular origin (Table 1).

**Table 1:** Selected identities from trypsin shaving experiment.

Category/Protein identity	Accession^a^		Exp1	Exp2	Exp3	Exp4
	NCBInr/Smp (SmGenes)	Clustered ESTs				
**Host proteins**						
haemoglobin α chain	AAA37783		•			
haemoglobin β-1 chain	P02088		•			
C3 complement	AAC42013				•	•
C4 complement	CAA28936		•	•	•	
CD44	CAA46880			•	•	•
**Membrane and membrane-associated**						
Annexin type IV	Smp_074140	Sm04814	•			•
Annexin type V	Smp_074150*			•		
Annexin type VI	Smp_077720	Sm03987	•	•	•	•
Calpain	Smp_157500	Sm11826			•	
Sm25	Smp_195180	Sm04760		•	•	•
Sm200	Smp_017730*	Sm03865	•	•	•	•
Proteoglycan-like	Smp_020550	Sm08105	•		•	
**Secretory and vesicular pathway**						
Potassium-channel inhibitor SmKK7	Smp_194830	Sm12916	•			
Endophilin (BAR domain)	Smp_003230.2	Sm04606	•	•	•	•
Endophilin (BAR domain)	Smp_163720	Sm00115*	•	•	•	•
**Gut proteases**						
Hemoglobinase (asparaginyl endopeptidase)		Sm00989		•		
Cathepsin B1 isotype 1 precursor	Smp_067060*	Sm01772*	•			

a Accession numbers for host proteins can be found at Pubmed http://www.ncbi.nlm.nih.gov/protein/Smp and Sm numbers are found at http://www.genedb.org/Homepage/Smansoni.

*Peptide hits common to more than one predicted protein.

Exp  =  Experiment.

The host complement proteins C3 and C4 and the leukocyte surface marker CD44 were released in two or more of the four replicate experiments. Host haemoglobin alpha and beta chains were also identified in one experiment, presumably an indication that regurgitation from the worm gut was occurring. A number of proteins, known to be associated with the tegument surface, were also released by the treatment. They included three phospholipid-binding annexins and the membrane protease calpain. Of the annexins, Smp_077720 was found in all four experiments and the others on two and one occasions, respectively; calpain was detected in only one experiment. The schistosome-unique proteins of unknown function, Sm200 and Sm25, were found on three and four occasions, respectively. The final membrane protein, not previously associated with the tegument, shows homology with cell surface proteoglycans. A pair of BAR-domain-containing endophilins was also released by the treatment in all four experiments while the putative potassium-channel inhibitor, SmKK7, was found on one occasion.

Although worm incubations were short-term, two known gut-derived proteases, asparaginyl endopeptidase and cathepsin B1 were detected. In addition, a total of 12 proteins with unknown function and localization were found (Tables S1 and S2), some containing domains, e.g. Ig-like and EGF-like, which may indicate a surface position. In spite of strenuous efforts to maintain worm viability, the trypsin treatment affected the integrity of the surface membranes to some extent, as evidenced by the appearance of seven cytoskeletal and 17 cytosolic proteins in the medium (Tables S1 and S2). Among the former, actin, fimbrin and severin have been proposed as constituents of the tegumental spines that reside immediately beneath the surface membrane complex. The more numerous cytosolic proteins, comprising glycolytic enzymes, chaperones, and antioxidants indicate the leakage of internal components in at least some of the parasites. Known tegumental surface transporters, ion channels, and enzymes (other than calpain) were conspicuous by their absence in the trypsin preparation.

### Enzymatic shaving using PiPLC

**Figure 1:**
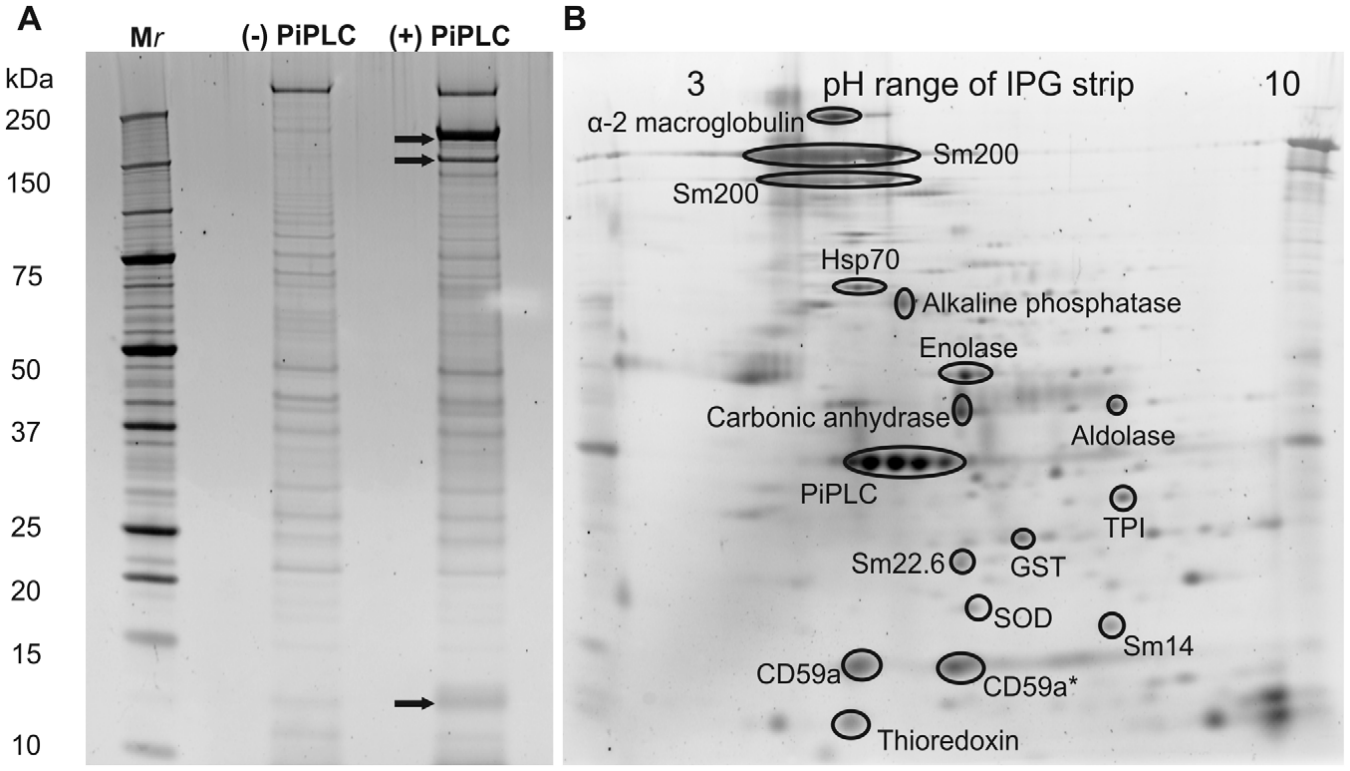
PiPLC-released proteins analysed by 1-DE and 2-DE. (A) Ten µg of proteins present in the culture supernatant +/− PiPLC were separated by 1-DE and the gel stained with SYPRO Ruby. The majority of the proteins were shared between the PiPLC-treated and non-treated samples. Only three bands (arrowed) were visibly enriched by the treatment, two of high molecular mass at approx. 200 kDa and a third at approx. 12 kDa. (B) Fifty µg of proteins present on the PiPLC- treated supernatant were separated by 2-DE and the gel stained in SYPRO Ruby. Labelled spots are those identified by mass spectrometry after Coomassie staining of the gel. The PiPLC protein cluster on the 2-DE map does not correlate with the same levels of PiPLC used for preparation of 1-DE gels as they were from different sources and exhibited differing specific activities. *Less acidic CD59 isoform, see Table S3 for peptide identification data.

The purpose of treatment with PiPLC was to release GPI-anchored proteins accessible to the externally applied enzyme in live worms; incubation with the enzyme for 1 h appeared to have no morphologically obvious deleterious effect. A 1-DE gel separation of material released by treated and control worms revealed a complex pattern of protein bands with M_r_ ranging from 10 to >250 kDa (Figure 1A). The two preparations displayed a strong similarity, with only three bands (arrowed) visibly enriched by the PiPLC treatment versus the control; two were of high molecular mass (approx. 200 kDa) while the other was located at the bottom of the gel (approx. 12 kDa). The PiPLC treatment released sufficient protein to permit a mini 2-DE separation (Figure 1B) for subsequent MS/MS analysis of gel spots. The analysis revealed the presence of proteins known to be GPI-anchored, such as ‘Surface protein’ (Sm200) and alkaline phosphatase. Protein orthologues of CD59 and carbonic anhydrase IV were novel features of this 2D map. The absence of gut proteases Sm31 and Sm32 was notable. The identities of other spots revealed the presence of cytosolic and cytoskeletal contaminants, including thioredoxin, fatty acid binding protein (Sm14), Sm22.6, enolase and triose phosphate isomerase (Table S3). The remaining spots on the SYPRO-stained gel (Figure 1B) were not detected by Coomassie staining so no identification could be assigned using single spot tryptic digestion.

**Figure 2:**
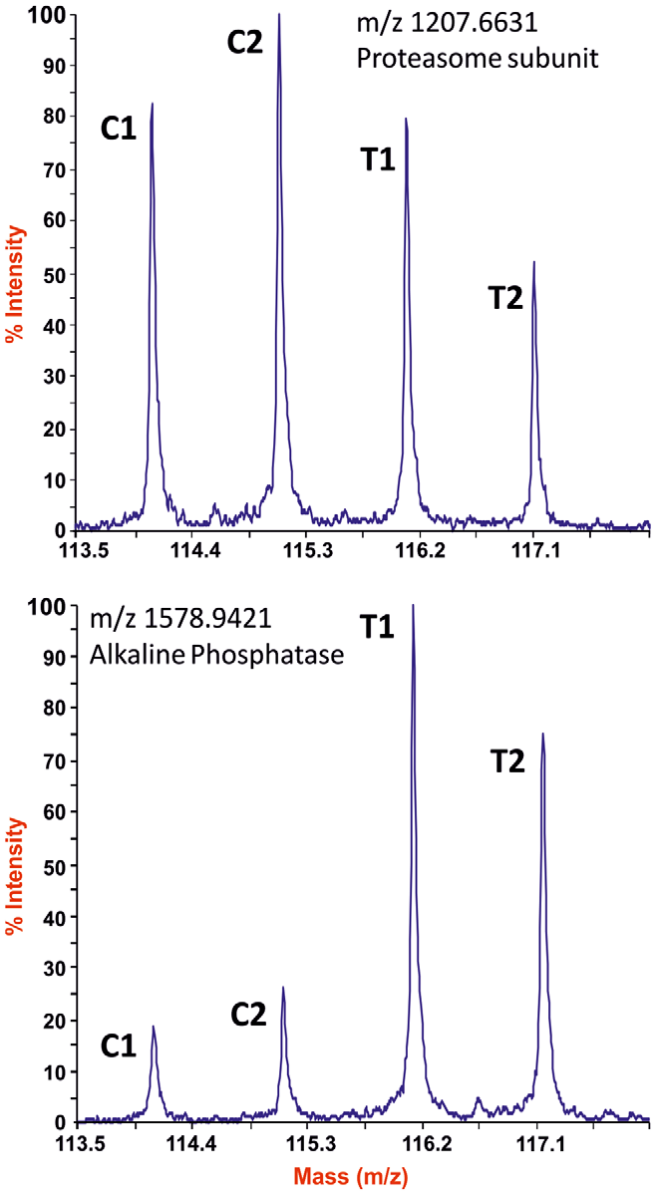
Discrimination between enzymatic enrichment and parasite leakage/secretion using iTRAQ tags. (A) Low *m/z* spectral region displaying the iTRAQ reporter ion peaks whose ratios revealed a non-GPI anchored protein. (B) Low *m/z* spectral region displaying the iTRAQ reporter ion peaks whose ratios revealed a GPI-anchored protein. C1 and C2 represent control samples labelled with the iTRAQ *m/z* 114 and 115 tags, respectively. T1 and T2 represent PiPLC-treated samples labelled with iTRAQ *m/z* 116 and 117 tags, respectively.

**Figure 3:**
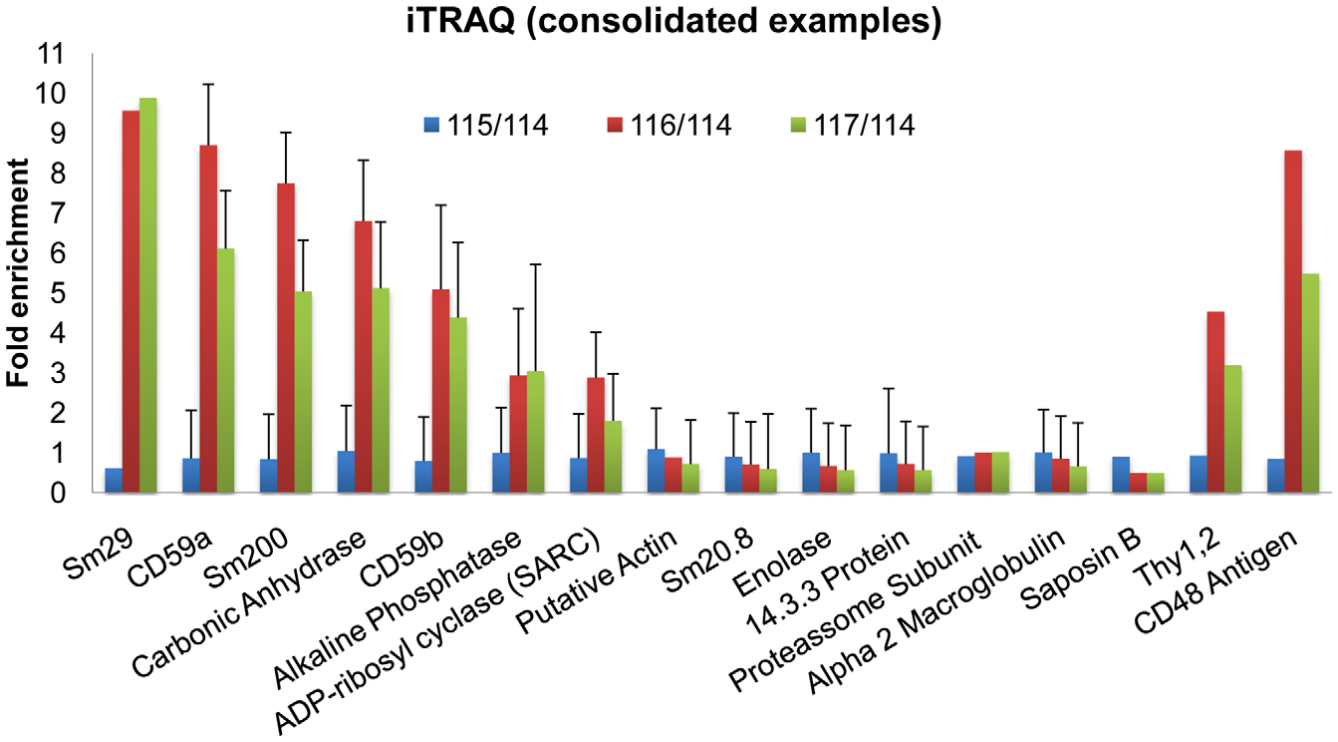
iTRAQ consolidated results. Sixteen out of 52 proteins identified in the iTRAQ experiment selected to provide a snapshot of the major findings provided by the PiPLC shaving approach. Fold enrichment on the y axis is plotted relative to the intensity of the *m/z* 114 C1 tag. The seven GPI-anchored proteins of parasite and the two of host origin are included. Note the equivalent levels of proteins representing cytosolic, cytoskeletal, secretory and gut contaminants present in both PiPLC-treated and control samples. C1, C2, T1 and T2 key as in Fig 2. Bars represent the standard geometric deviation for iTRAQ ratios associated to protein identities obtained with at least 3 peptide fragmentations. iTRAQ ratios and protein identities obtained from fragmentations of 1 and 2 peptides are reported based on their significant Mascot expect score (<0.05) in three independent iTRAQ experiments.

As proteins were released into the culture medium during the 1 h incubation irrespective of whether PiPLC was present or not, we used the iTRAQ technique to determine the degree of enrichment due solely to the enzymatic shaving. Within an individual labelling protocol, splitting both the control and treatment samples provided technical replicates. After LC separation of the tagged peptide mixture (Figure S1B), fragmentation spectra of the most abundant peptides were generated, each containing signature peaks for the four reporter tags (Figure 2 and Figure S2). In most instances the fragmentation spectra yielded four peaks of approximately equal area (Figure 2A). The 115/114 (C2/C1) ratios approximated to unity, indicating approximately equal protein contributions of the two control samples to the iTRAQ mixture. However, the majority of 116/114 (T1/C1) and 117/114 (T2/C1) ratios were almost invariably less than one, sometimes significantly so (Figure 2A and Figure S2). We have no reason to believe that the control and treatment incubations differed except in the addition of enzyme, and so we must attribute the ratios of less than unity to a dilution effect on the background proteins in the treated samples. This dilution resulted from the addition of PiPLC plus the extra proteins released from the surface by its action. In a minority of fragmentation spectra the four peaks representing the reporter tags were not of approximately uniform area (Figure 2B and Figure S2). The reporter ion peaks from the treated samples were from 2 to 10 fold greater intensity indicating enrichment of the parent peptide by the PiPLC treatment. A total of 52 identities was obtained from fragmentation of the tagged peptides (Figure S2 and Tables S4 and S5) ranging from 1 to 15 per identity, primarily of cytosolic or cytoskeletal origin. The PiPLC-enriched proteins of parasite origin were, in descending order of abundance, Sm29, CD59a, Sm200, carbonic anhydrase, CD59b, alkaline phosphatase and ADP-ribosyl cyclase (Figure 3). In addition two host proteins, CD48 and CD90 (Thy1.2), were also enriched by PiPLC treatment. Proteins of known gut origin, α2-macroglobulin and saposin B, were present in control and treated samples in equivalent amounts indicating similar regurgitation of gut contents over the one hour incubation. Equivalent amounts of cytosolic (e.g, enolase, 14-3-3) and cytoskeletal proteins (e.g, actin, Sm20.8) in control and treated samples (Figure 3 and Table S4) indicated a certain degree of surface damage, but not inflicted by the PiPLC treatment. Although not enriched by PiPLC hydrolysis, because they lack the GPI-anchor, three other proteins are worth noting. Two of these, CD63/tetraspanin (TSP-2) and annexin IV (Smp_074140) were already known to be associated with the tegument surface and the third is an 8 kDa low molecular weight protein (LMWP). This last protein, present in a range of trematodes [10], has a signal peptide and so may be a true secreted protein released along with the membranocalyx; its status as a tegument surface protein needs to be confirmed.

### Enzymatic shaving using PLA2

Unlike the other two enzymatic treatments PLA2 had a dramatic effect on worm appearance and viability. Moderate concentrations of PLA2 produced visible worm damage and death, whereas greater dilutions had little selective effect in removing known tegumental surface components whilst still causing leakage of cytoskeletal and cytosolic components (Table S6). The known tegument surface proteins released were Sm29, LMWP and dysferlin. The PLA2 approach was therefore discontinued.

### Localization of host membrane proteins

**Figure 4:**
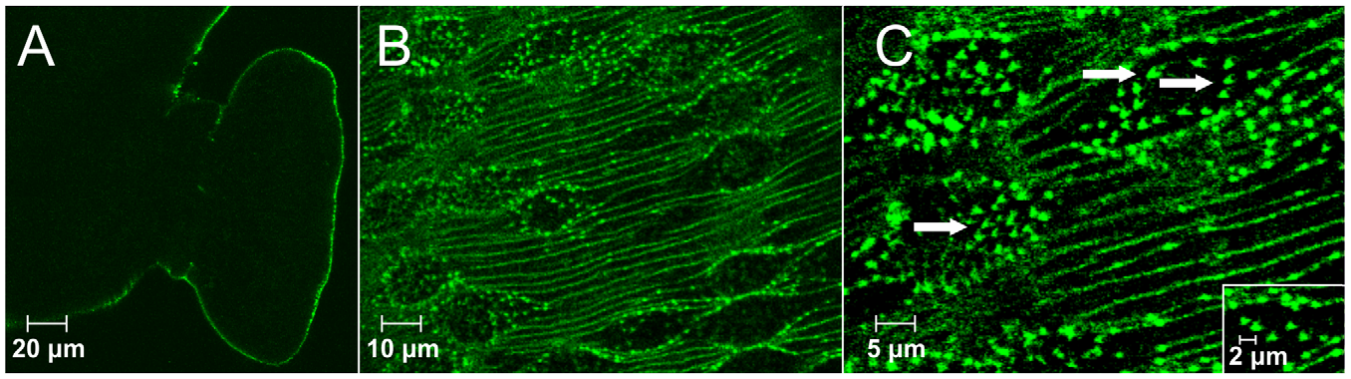
Immunocytochemistry of host CD44 on the *S. mansoni* tegument surface. (A) OTC-embedded parasite sections and (B/C) freshly perfused live parasites were labelled with rat anti-mouse CD44. In worm sections, CD44 staining was confined exclusively to the parasite surface. Figure (A) represents the CD44 staining of the tegument in the ventral sucker region. (B) Slice from a Z-stack of the male tegument after CD44 staining of live worms. (C) Higher magnification of (B) showing a striking inverted V pattern for CD44 staining being mostly concentrated at the tip of the spines (arrowed).

Confocal microscopy of adult worm sections revealed that host CD44 was confined entirely to the tegument with no staining of internal structures (Figure 4A). Examination of a Z-stack through the tegument of an intact male worm revealed that the pattern of staining was not uniform, being concentrated on numerous, parallel, transverse ridges and especially the spines on the dorsal tubercles (Figure 4B). On close inspection, the individual spines had a definite inverted V appearance the most intense staining being at the tip (Figure 4C). Antibodies to CD48 and CD90 failed to detect their respective targets on the worm surface.

### Transcripts for tegument surface proteins shared between adult worms and lung schistosomula

Our in-house EST database was interrogated for the occurrence of transcripts in lung schistosomula encoding the proteins of interest released from adult worms by the enzyme treatments. Although it is difficult to make inferences about abundance as some data were obtained from normalised libraries, nearly all transcripts were represented in both larvae and adults in roughly similar numbers (Table 2). The exceptions were the proteoglycan (5L and 0A) and ADP-ribosyl cyclase (0L and 1A). Transcripts for calpain and Sm200 were particularly abundant, whilst the largest number recorded (63) was for one of the CD59 orthologues in the lung schistosomulum. Based on the transcript evidence we tentatively conclude that the most exposed tegument surface proteins of adults are also likely to be present on the surface of the migrating lung schistosomulum.

**Table 2:** Number of transcripts for tegument surface proteins shared between lung schistosomula (L) and adult worms (A).

Protein	Gene model	L	A
Annexin type IV	Smp_074140	4	3
Annexin type V	Smp_074150	1	8
Annexin type VI	Smp_077720	7	6
Calpain	Smp_157500	17	21
Sm25	Smp_195180	2	2
Sm200	Smp_017730	11	23
Proteoglycan-like	Smp_020550	5	0
Endophilin (BAR domain)	Smp_003230	2	13
Endophilin (BAR domain)	Smp_163720	4	8
Sm29	Smp_072190	2	6
CD59/Ly6 (CD59a)	Smp_019350	2	4
CD59/Ly6 (CD59b)	Smp_105220	63	3
Alkaline phosphatase	Smp_155890	4	10
Carbonic anhydrase	Smp_168730	2	3
ADP-ribosyl cyclase (SARC)	Smp_025830	0	1
Tetraspanin TSP-2	Smp_181530	6	15

### Mapping of peptide hits for Sm200

Only one tegument surface protein, Sm200, was released by both the trypsin and PiPLC treatments of live worms. The difference between the two was that PiPLC released the entire GPI-anchored molecule for subsequent trypsinisation as a separate step. Conversely the trypsin treatment released peptide fragments from Sm200 molecules only during the incubation period, which were then recovered using reversed-phase chromatography. Mapping of the peptide hits onto the primary amino acid sequence revealed a very different pattern of distribution (Figure S3). A total of 15 peptides, with a significant Mascot score, was identified for the PiPLC-released protein, distributed throughout the entire molecule. In contrast only six peptides were identified for the trypsin-released protein and four of these were clustered towards the C-terminus of the protein, i.e. the region located nearest to the GPI anchor. This may indicate that only part of the Sm200 protein is accessible to trypsin in the live worm. In this context, the programs NetNGlyc and NetOGlyc both at http://www.cbs.dtu.dk/services/ predicted five N (scores >0.6) but no O-glycosylation sites on the amino acid sequence coding for Sm200. It is notable that four of the five potential N-linked sites are located furthest from the GPI anchor site in the N-terminal region where few peptides were identified following trypsin treatment of the live worms. Thus it is plausible that attached N-glycans protect the native Sm200 polypeptide chain in situ on the worm surface from trypsin attack.

## Discussion

Our previous biotinylation studies have provided insights into the disposition of proteins in the complex molecular structure of the schistosome tegument surface. In the present study we have taken an alternative approach to obtain information about the location of tegument components. This involved shaving the surface of live adult worms with selected enzymes to release accessible proteins, and their identification by proteomics. It is axiomatic in this approach that the integrity of the worm surface is not compromised by the treatment. Our results indicate that trypsin and PiPLC largely fulfilled this criterion but PLA2 did not. We must assume that this last enzyme, via its attack on the lipid bilayers, rapidly caused generalized erosion of the surface membranes and loss of integrity. We shall therefore consider only the results of the trypsin and PiPLC treatments to make inferences about tegument surface organization. In addition, the male parasite has at least two times the surface area of the female, assuming all surfaces are accessible to the enzymes. If the gynaecophoric canal is effectively sealed, then the male dorsal surface would have contributed the bulk of released protein.

It was inevitable that the *in vitro* culture of live worms resulted in the contamination of the enzyme-released proteins by gut content. Thus the detection of proteins such as α2-macroglobulin, saposin B, and hemoglobinase (asparaginyl endopeptidase) in the two enzymatic shaving experiments was to be expected. Conversely, even with the most careful handling of parasites including their recovery from mice by perfusion with culture medium, the presence of cytosolic and cytoskeletal proteins must be attributed to some degree of worm damage. In the present study, we used the iTRAQ labelling technique on test and control samples to discriminate enzymatic enrichment from normal secretion or protein leakage due to invisible damage. This approach successfully overcame the dominance of abundant peptides from contaminating cytosolic and cytoskeletal proteins to highlight the few proteins released by PiPLC treatment. Nevertheless, one must bear in mind that as enzyme accessibility has not been addressed in this investigation, caution should be exercised when considering the fold enrichment found for GPI-anchored molecules. In this regard, it is possible that a higher fold enrichment for a given protein may only indicate a more exposed/accessible location at the parasite surface rather than imply protein abundance.

A major finding of our study is that seven parasite proteins can be detached from the surface by treatment with PiPLC and enriched compared to control incubations. From this we infer that each is inserted into the parasite surface by a GPI-anchor. As these anchors are invariably located at the C-terminus of such proteins we conclude that the extraneous 30 kDa PiPLC enzyme is able to access the anchor in proximity to a lipid bilayer. Whether this bilayer is the secreted membranocalyx or the underlying plasma membrane is problematic. Access to the latter would mean that the digesting enzyme had to pass through the protective membranocalyx. One possible inference is that the seven GPI-anchored proteins are inserted into the membranocalyx. For carbonic anhydrase this seems unlikely because of its assumed function in regulating acid-base balance at the parasite surface. It would be best placed to do this if located between the two lipid bilayers so that, by analogy with the erythrocyte, CO_2_ generated inside the worm could be converted to HCO_3_^−^ for diffusion into the bloodstream. Indeed, the reverse diffusion of a chloride ion through the membranocalyx would be required to balance the charge, bringing it into close proximity with the anion transporters, which our previous studies have shown are present in the tegument plasma membrane [6]. The gene model for carbonic anhydrase is incomplete, lacking both the N-terminal exon encoding the signal peptide and the C-terminal exon(s) encoding the site for the attachment of a GPI-anchor. However, its enrichment by PiPLC treatment of live worms attests to the presence of a GPI-anchor. Two isoforms of CD59 orthologues, identified by possession of the CCxxDxCN motif, were also highly enriched by the PiPLC treatment, implying their outer location at the surface. These proteins are potentially significant because the human CD59 protein protects self-cells against complement fixation by blocking formation of the C5 to C9 membrane attack complex [11]. A similar role for the two schistosome molecules is very attractive, as a component of the parasite's mechanisms of immune evasion. It is of note that in the *S. mansoni* genome there are four additional gene models encoding CD59 orthologues, which could also be tegument-associated [12]. These CD59 orthologues in the tegument surface are better candidates for inhibition of complement fixation than the SCIP-1 protein [13], subsequently identified as paramyosin [14], [15].

The protein most highly enriched by PiPLC treatment was Sm29 that has no known function. It was originally selected by a bioinformatic search as a protein with a single membrane spanning domain [16]. It was identified in the final pellet after differential extraction in a compositional analysis of the tegument membranes [6] and was accessible to biotinylation in live worms [7]. Subsequent confocal microscopy revealed its association with the tegument although technical issues do not allow a firm conclusion about its precise location [17]. Our PiPLC shaving results demonstrate both its peripheral location and the fact that it is GPI-anchored, the latter prediction also made by big-PI Prediction server (http://mendel.imp.ac.at/gpi/gpi_server.html). The peripheral location of Sm200, again with no known function, was also revealed by PiPLC shaving. This protein first cloned by Hall et al. [18] was already known to be GPI-anchored and located in the tegument surface [19], [20]. Surprisingly therefore it was not found in compositional analysis of the tegument membrane [6], but was accessible to biotinylation in live worms [7]. These observations suggest that whilst definitely surface-attached it is readily lost during the processing of tegument membranes by differential extraction for MS analysis, a feature that may be linked both to its size and GPI-anchor. Moreover, Sm200 has recently been identified in circulating lipoprotein particles from the blood of schistosome-infected humans, confirming its turnover into the vascular environment [21].

The two remaining GPI-anchored proteins shown to be enriched by iTRAQ labelling after PiPLC treatment were alkaline phosphatase and ADP-ribosyl cyclase. The former is well characterized and was previously shown to be GPI-anchored in schistosomula [22]. It has been used as a membrane marker in the development of methods for tegument surface isolation [5]. It was identified by compositional analysis of the tegument surface membranes in the urea/thiourea/CHAPS/sulfobetaine (UTCS) fraction and insoluble pellet [6], and is accessible to biotinylation in live worms [7]. Its presence in the UTCS fraction indicates that it is, at least partially, loosely associated with the surface membranes i.e not membrane spanning. The recently characterised ADP-ribosyl cyclase was also shown to be GPI-anchored and localized to the outer tegument of the adult schistosome [23]. Both schistosome enzymes must be able to access their substrates in live worms but their precise function at the tegument surface remains to be established. In the case of ADP-ribosyl cyclase, a role in calcium mobilization has been proposed [23] but alternatively it could function in immune evasion by regulating ecto-NAD+ levels, thereby reducing substrate availability for CD38- and CD157-mediated effector functions of lymphocytes. Corroborating the identification of GPI-anchored molecules using the iTRAQ technique, four out of the seven proteins assigned as GPI-anchored were also detected by our 2-DE analysis. These were Sm200, alkaline phosphatase, two isoforms of CD59 orthologues and carbonic anhydrase. A definite proof of their enrichment due to PiPLC's activity is the fact that these molecules are underrepresented and are not easily identifiable in 2-DE maps produced using soluble worm preparations [8] or membrane-extracted proteins from crude or differentially-extracted *S. mansoni* tegument [6].

Use of the iTRAQ technique to identify proteins enriched by trypsin treatment was not possible because the released material was already partially digested. This prevents accurate quantification of protein for labelling and therefore the trypsin shaving fraction was subsequently processed as a peptide digest. We have taken as a reference of cytosolic/cytoskeletal contaminants in the trypsin shaving, the number and diversity of those proteins released during 1 h incubation at 37°C in the absence of added enzyme. When we compared the number of proteins that could indicate leakage or vomitus with the number of proteins in the trypsin-treated parasites, we observed we had less contamination, most likely due to a shorter incubation time, 30 min. In addition, we are assuming that as trypsin should not be able to permeate the parasite tissues, the peptides originating from membrane proteins are likely to represent the most exposed/accessible domains at the parasite surface. We therefore focus only on (1) membrane and membrane-associated, (2) vesicular pathway and secreted, and (3) host proteins. The peripheral location of Sm200 was again confirmed but it was the only one of the seven PiPLC-released proteins that was also released by trypsin. This suggests that the rest may be protected from proteolysis, potentially by their N and/or O-linked glycans or by a sequestered location. The release of three annexins by the trypsin treatment indicates their superficial location. One (Smp_077720) was previously detected by both biotinylation and compositional analysis and two are new to this study (Smp_074140 and Smp_074150). The known phospholipid-binding properties of this group of proteins means that they could have a role in promoting adhesion of the membranocalyx to the plasma membrane, acting like a molecular ‘velcro’ via the four binding domains that each possesses. More recently, certain annexin isoforms have been implicated in immunomodulatory functions such as the resolution of inflammation [24]. They are able to interact with receptors on the surface of leukocytes to control apoptosis and their clearance by macrophages. The existence of such a process at the surface of the tegument, mediated by schistosome annexins, accords with the absence of leukocyte binding to adult worms in vivo [25]. However, a comparison of the orthologies of the schistosome and human annexins is difficult because of the evolutionary distance. Thus, BLAST searching of the schistosome sequences against the NCBInr database reveals the closest homologues of Smps 077720, 075150 and 075140 are human annexins A13, A7 and A8 respectively, not the A1 isoform that has been most implicated as an anti-inflammatory agent. The conjecture will only be resolved by expression of the schistosome annexins for assays of function.

The presence of Sm25 as a tegument surface protein has a chequered history. It was proposed as a vaccine candidate because anti-Sm25 antibody levels correlated with protection in mice vaccinated with a crude tegument membrane preparation [26]. It was then cloned and designated as an N-glycosylated integral membrane protein [27] but later characterized as a palmitoylated protein with the implication that it was on the cytosolic leaflet of the plasma membrane [28]. Further immunocytochemical studies suggested it was distributed throughout the tegument syncytium but not associated with the surface membranes [29] and it could only be biotinylated when parasites were permeabilized by Triton X-100 [30]. It was not found in either our compositional or biotinylation studies on the tegument surface. The removal of Sm25 from live worms by trypsin, when so few other membrane proteins are released, suggests a unique accessibility. The proteolytic enzyme calpain was previously identified at the tegument surface by both compositional analysis [6] and biotinylation [7]; its potential as a vaccine candidate has already been exploited [31], [32]. The final member of this group is a heparin sulphate-like proteoglycan which has not been identified in any previous studies and merits further investigation. In the secreted protein category, this is the first report of SmKK7 release from the tegument surface; it has previously only been reported from cercarial secretions [33]. The detection of a pair of BAR domain proteins in all four experiments after trypsin treatment is intriguing since these banana-shaped molecules located on the cytosolic surface of plasma membranes function to promote membrane curvature in exo- and endocytosis [34]. This suggests that the trypsin may be entering the fine tubules located at the base of tegument pits where multilaminate vesicle fusion with the tegument plasma membrane occurs [35], [36]. That conclusion would place Sm25 and the proteoglycan in a similar location where constituent proteins are poorly protected by overlying membranocalyx, perhaps loosely analogous to the relationship of the flagellar pocket of trypanosomes [37] to variant surface glycoprotein export.

The release of complement C3 from the parasite surface by trypsin treatment is consistent with our previous biotinylation study [7]. We can now add C4, its precursor in the complement cascade, but not the C5 to C9 components of the membrane attack complex, suggesting complement fixation but then inhibition of the cascade. The failure to detect any immunoglobulins, when murine IgM, IgG1 and IgG3 heavy chains were biotinylated [7], is puzzling as they would be expected to initiate complement fixation. However, there is evidence that these proteins are resistant to trypsin degradation, particularly under the low concentration conditions employed in our experiments [38]. In fact, aiming to preserve worm viability, trypsin was used at a concentration approximately 100 times lower than that typically employed for stripping adherent cells from culture flasks. A positive consequence of such a gentle shaving treatment on live worms is justified by the reasonable number of genuine membrane proteins that were identified. In contrast, a previous study on *S. bovis* with trypsin treatment, used methanol-fixed parasites [39] and found an overall higher number of protein identifications. However, membrane-associated and/or integral proteins were poorly represented, and the results are not comparable with our approach using live worms.

Our enzyme shaving experiments extend the list of host proteins known to be firmly associated to the parasite surface. The release of CD48 and CD90 (Thy1.2) by PiPLC confirms their possession of GPI-anchors and most likely accounts for their transference from the host (leukocytes) in the same manner as host glycolipids transfer from erythrocytes through a process currently termed cell-painting [40]. Supporting this finding is the demonstrable ability of purified Thy-1to reincorporate into the plasma membrane of murine Thy-1- cells directly from aqueous suspension and without the use of detergents [41]. However, failure to detect the presence of both CD48 and CD90 by immunocytochemistry indicates their relative paucity. The detection of host CD44 is more surprising since it is an extracellular protein anchored in the membrane of a range of cell types by a single transmembrane domain [42]. However, the intense staining of the tips of spines on the dorsal tubercles, the point of contact between the worm and the vascular endothelium during peregrination around the portal vasculature, suggests that the transfer occurs when the apposing membranes of tegument and endothelium are pressed together. The observations on CD44 staining are a testament to the acquisitive properties of the schistosome surface.

Our studies on the surface accessibility of tegument proteins are relevant to the development of schistosome vaccines. In mice immunized with attenuated cercariae challenge schistosomula are eliminated in the lungs by inflammatory foci that block their onward migration [43]. Both priming of the immune response [44] in skin-draining lymph nodes and the pulmonary effector responses appear to be mediated by proteins on the schistosomular tegument surface [45]. Although we have characterised surface proteins on the adult worm, the presence of the encoding mRNAs in the lung schistosomulum provides support for the suggestion that those same proteins are exposed on the larval tegument surface. In this context Sm29 has already been used successfully as a vaccine candidate [17]. We therefore suggest that the other six PiPLC-anchored proteins would also repay investigation as putative vaccine candidates, especially Sm200 because it was also removed by trypsin. Indeed, the presence of a GPI-anchor may make proteins in this group more prone to detachment from the surface for transcytosis across the pulmonary capillary endothelium, processing by accessory cells and presentation to reactive CD4+ Th1 cells in the lung parenchyma. To this list we can add the three annexins released by trypsin and LMWP that may be a tegument secretion, noting also that calpain is a proposed vaccine candidate [31]. It is conceivable that a cocktail of these proteins would elicit strong protection against intravascular migrating schistosomula, especially if targeted to the lungs, whereas immunisation with a single protein has only modest success [46].

## Supporting Information

Figure S1 Trypsin and PiPLC shaving experiments. Peptide Chromatograms at 214 nm.
(0.13 MB PDF)

Figure S2 Protein identities and iTRAQ ratios from PiPLC shaving approach. Bars represent the standard geometric deviation for iTRAQ ratios associated to protein identities obtained with at least 3 peptide fragmentations. iTRAQ ratios and protein identities obtained from fragmentations of 1 and 2 peptides are reported based on their significant Mascot expect score (<0.05) in three independent iTRAQ experiments.
(0.53 MB PDF)

Figure S3 Sm200 tryptic peptides identified using trypsin and PiPLC shaving of live parasites. Putative N-linked glycosylation sites are indicated in red.
(0.01 MB PDF)

Table S1 Other identities from trypsin experiment.
(0.03 MB XLS)

Table S2 Trypsin shaving peptide data.
(0.10 MB XLS)

Table S3 Identities from 2-DE gel of PiPLC-released proteins.
(0.03 MB XLS)

Table S4 PiPLC shaving identities/iTRAQ ratios.
(0.04 MB XLS)

Table S5 PiPLC shaving peptide data.
(0.06 MB XLS)

Table S6 PLA2 shaving identities and peptide data.
(0.03 MB XLS)
