# Supplementary material for: Enzymatic Shaving of the Tegument Surface of Live Schistosomes for Proteomic Analysis: A Rational Approach to Select Vaccine Candidates
Source: PLoS Negl Trop Dis. 2011 Mar 29;5(3):e993. doi: 10.1371/journal.pntd.0000993 (PMC3066142; doi:10.1371/journal.pntd.0000993)

A – Peptide chromatogram of a representative Trypsin shaving experiment. See Materials and Methods for nanoHPLC separation conditions.

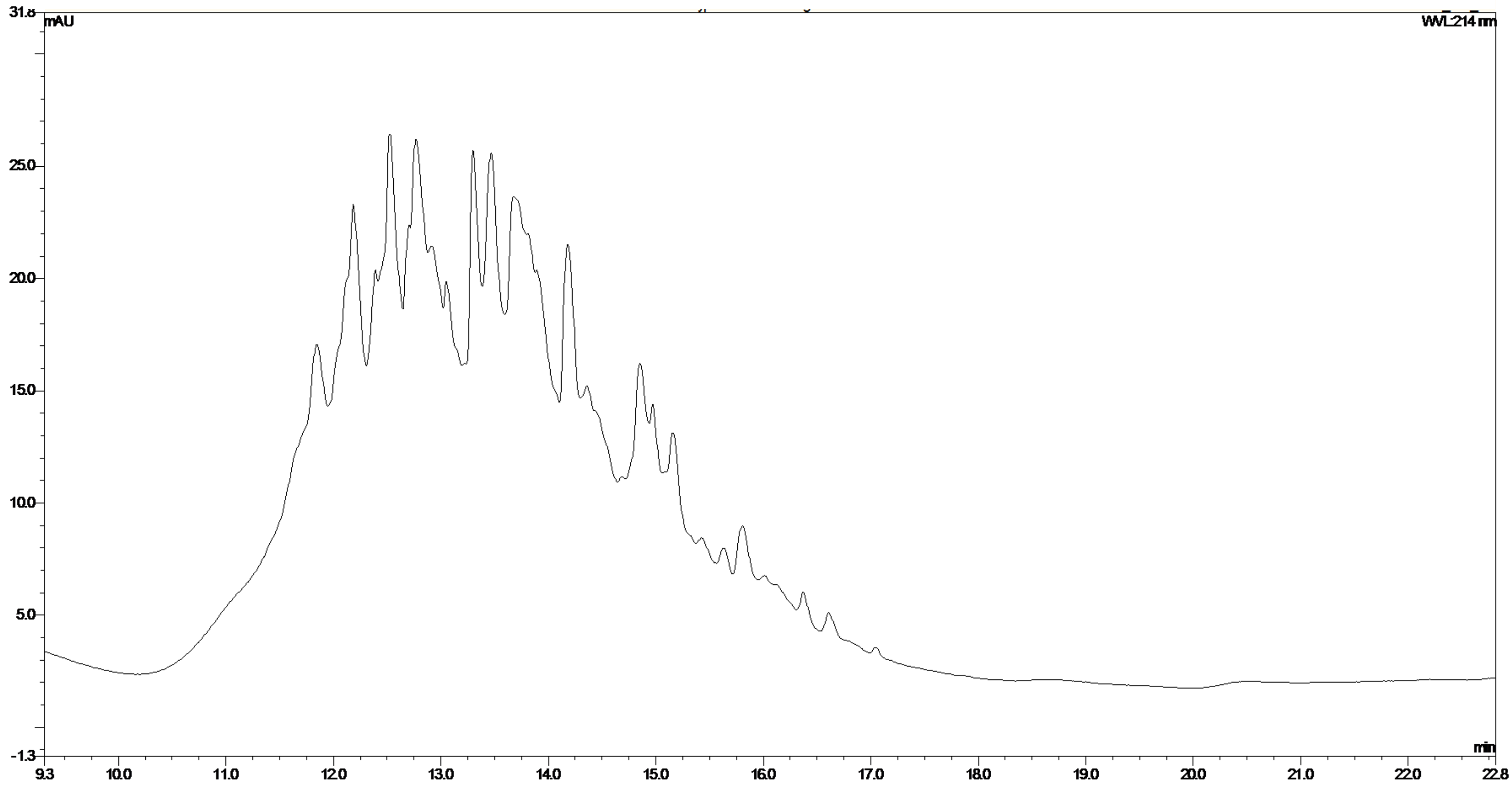

B - Peptide chromatogram of a representative PiPLC shaving / iTRAQ experiment. See Materials and Methods for nanoHPLC separation conditions.

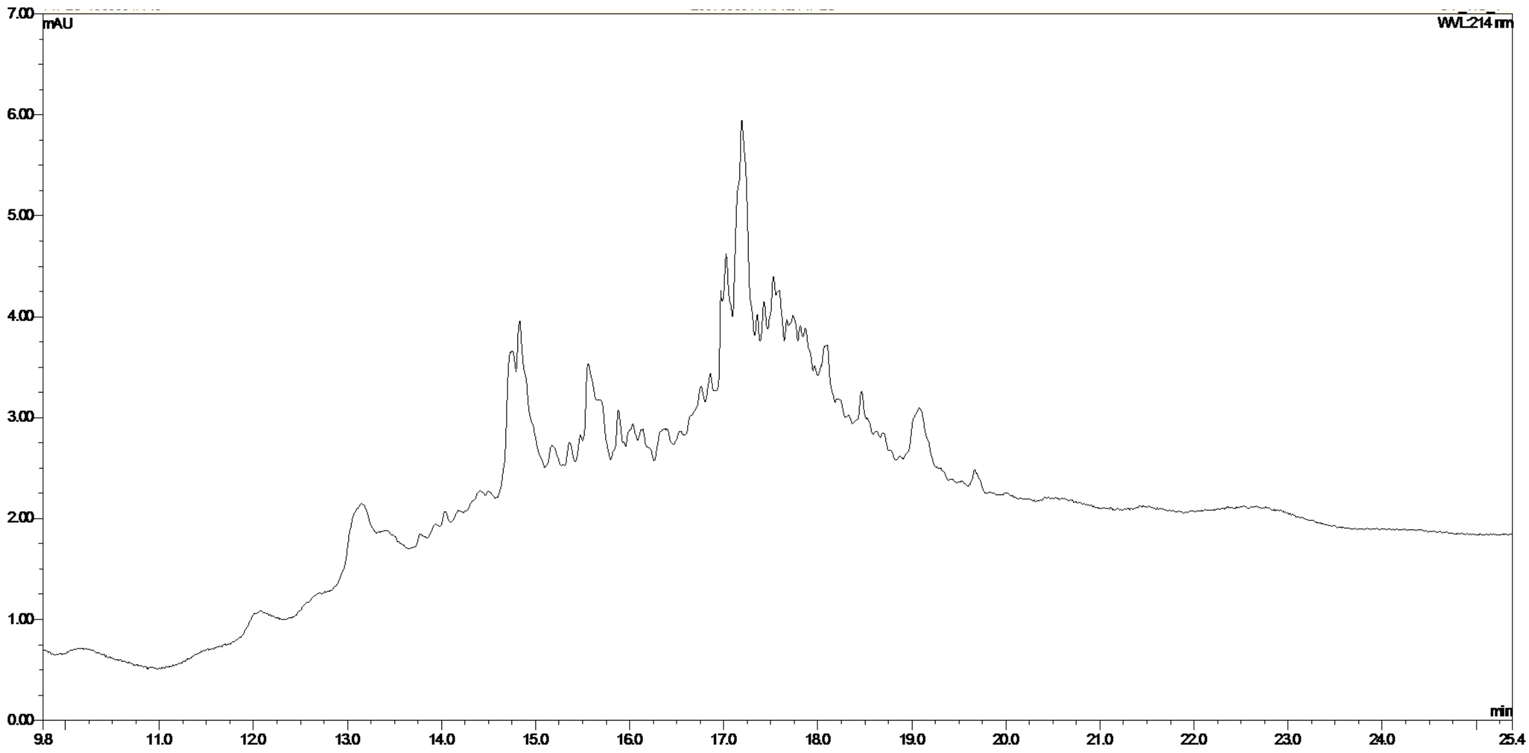

Supplement: Figure S1 — Trypsin and PiPLC shaving experiments. Peptide Chromatograms at 214 nm. (0.13 MB PDF) [file pntd.0000993.s001.pdf]
