# Supplementary figures and images for: Enzymatic Shaving of the Tegument Surface of Live Schistosomes for Proteomic Analysis: A Rational Approach to Select Vaccine Candidates
Source: PLoS Negl Trop Dis. 2011 Mar 29;5(3):e993. doi: 10.1371/journal.pntd.0000993 (PMC3066142; doi:10.1371/journal.pntd.0000993)

# iTRAQ labelling and protein identifications

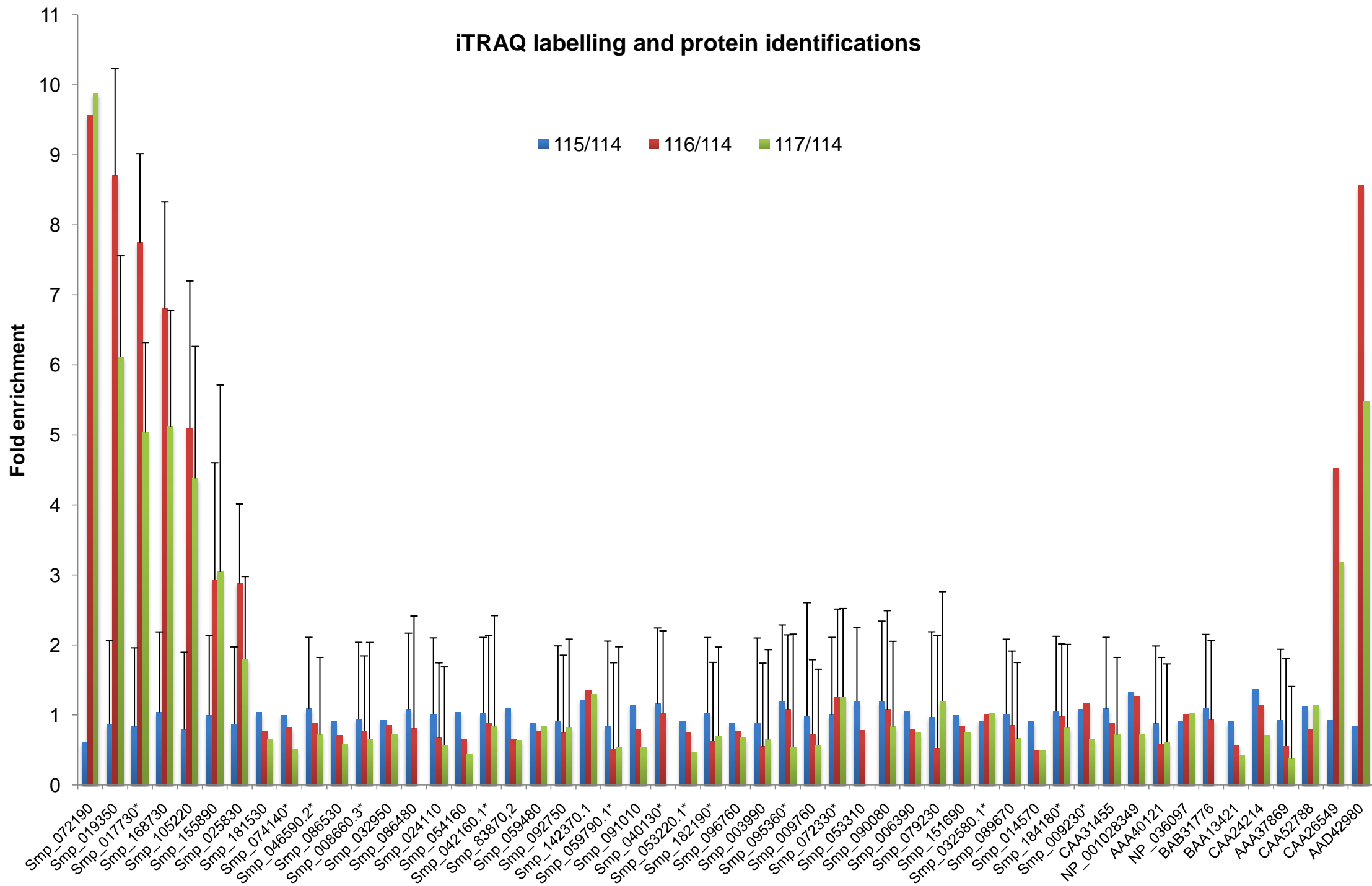

Supplement: Figure S2 — Protein identities and iTRAQ ratios from PiPLC shaving approach. Bars represent the standard geometric deviation for iTRAQ ratios associated to protein identities obtained with at least 3 peptide fragmentations. iTRAQ ratios and protein identities obtained from fragmentations of 1 and 2 peptides are reported based on their significant Mascot expect score (<0.05) in three independent iTRAQ experiments. (0.53 MB PDF) [file pntd.0000993.s002.pdf]
