# Supplementary material for: Enzymatic Shaving of the Tegument Surface of Live Schistosomes for Proteomic Analysis: A Rational Approach to Select Vaccine Candidates
Source: PLoS Negl Trop Dis. 2011 Mar 29;5(3):e993. doi: 10.1371/journal.pntd.0000993 (PMC3066142; doi:10.1371/journal.pntd.0000993)
Supplement: Figure S3 — Sm200 tryptic peptides identified using trypsin and PiPLC shaving of live parasites. Putative N-linked glycosylation sites are indicated in red. (0.01 MB PDF) [file pntd.0000993.s003.pdf]

Peptide hits for Sm200 (Smp_017730) - PiPLC ( ) versus Trypsin shaving ( ). Peptide common to both enzymatic treatments ( ) and predicted N-linked glycosylation sites

MYFLQLFLFLFTSVCNTLSDLHSNNVVDIKAHDYKLLTKILAARQLQDLFDNDKNTHGLFHAELNQKVYLIV
DLGGLYQVSSVEITSDEPENLKQTISIGYGFDKNTTSLFGSLYNCTYQQTSTLCIPACSQYDNSR.TGFVGN
SLLWSFQSDK.EGWTRIYDLIIRGTPFDENRSMKNNLDDITLFKPYVDHLVPAESMESCNGLLWHLNDESS
VTESSALAATDDRNVTIYFGK.TYSVTSVHFVTSK.RDDMPQEYTLHFNGMESLTKIINLQNDCTLTASSDND
GANFKEYNCPTTDLESYTFDYVTVNVKGLYKLHVYGLPFHYPKIQIIPSKEDDKTIMVDKNVLQISCIAQSC
NSTSNVLLDVDDSYRRSRSSDKSCPMNGHIVRCEYLNLFRLSTSPNDDNNQK.IQLTNQGIINTEFSEIMTE
LR.IIQSNAEK.LIVTMPRTHQYYSQYECSCQATDNKKSQLFSLSTNLKPTDFDQDFIFQTNYTTIFADQTIES
HVGFIELPENEHESYFKLNIIGYSLLNDVQIGVVFVEGGQAGSRTNANIEEISVQAFPGINVGSDLWSIPVYK
YELQDVWPDSVDDSVITVTWSMPKALSATKSPSVIRDIFR.ALIITSSGVNTIR.AWVWQRDSHLLDIR.AYHQ
LDENNVDSQLK.ILTLQRSGCSPSESEDEVVASVQLK.DGQCSTDNNDIITCTR.TIHGQIIQFK.LNNPSTSDV
YK.LYMKSDGVEDNVESTSSIDLVTSGSLGETVKEDIKGAGLSLTVEGIHHNHETQETELDVAVHIASKVISD
NIACRPTYLLLEFIEPNIETLKSRVSSKQTMFRIKLPSNQKEINLKMQLSIGSVDPTQSEATTNQSIAFQNPFY
IPTDIKVDAENQLIQWFGLPTIFNNLLHHYETKLSGLPKACEQASEFNLPITQQEIDNGTIYRVNLK.NIPDPTI
TK.NGLAIDYNFKVTPVFKGIDGKSITMGTSSDIRFSTGR.TGQTDLKAPTSGR.YYSLQVQVRPSQIPSCLNL
ETLNTQFILR.VIGEVDEYPDYIK.QVNYVPITMK.TTETLNDKNNHVK.LYKIENLLPGRRYELQEVHVQTEEV
FISPGERVVINCTGSVGPNDTSQKSLEWKLFDGGRLPDGSRSLKTQEAQSGPLWYAMESLIFDPVNKQHG
GVYACFIRPSILELMNKPTELHKVTVTVSDLEVDINSK.IVEFGEK.IIITCRTASPGQLDWMLPSGEKVEIMNE
MKSDDDNDDQPYTIKDENDDVKLSIKLIIPK.VNLNKVGK.YTCLHSPSNNKQTFSLKMKEVIKLVKSPESSD
KPGKTLILDCTANLGNLHQSVVWYK.RPNSNSPWLEITEAIQTIEHITIQQK.NPEDTLSSGVWLSELK.VK.NS
PGIIGEFMCTIQNIQTTMNIER.METGSIMTNDNDFSKITHATIKVSLKSVLKILTPIKLENGQISVHCQGYPAH
SKDR.LQWVYIPLNTDNK.SDKVITIVHSNPKDEENDQESDGNTKSLDEKEIDEIVSLAFQLTDSIPATWPGS
TGPQQLVQSGLIEQEHQPKQMYTAERLSLIFDSKYAEK.VADGILSCR.YVRPKGILPMDSDEAAESLSKVTI
PETNDDSDEILEKSEIPMKTLLDAK.QGDDNDNLSILK.SSLNEEPDEVQSDDKKNNTEKKSISLLLYMNLMA
LMIIFMRN
